# Optimization of the Loading of an Environmentally Friendly Compatibilizer Derived from Linseed Oil in Poly(Lactic Acid)/Diatomaceous Earth Composites

**DOI:** 10.3390/ma12101627

**Published:** 2019-05-17

**Authors:** Lucia Gonzalez, Angel Agüero, Luis Quiles-Carrillo, Diego Lascano, Nestor Montanes

**Affiliations:** 1 Technological Institute of Materials (ITM), Universitat Politècnica de València (UPV), Plaza Ferrándiz y Carbonell 1, 03801 Alcoy, Spain; lugona@epsa.upv.es (L.G.); guanche.ar@gmail.com (A.A.); nesmonmu@upvnet.upv.es (N.M.); 2 Escuela Politécnica Nacional, Quito 17-01-2759, Ecuador; diegol-zn@hotmail.com

**Keywords:** maleinized linseed oil MLO, poly(lactic acid), diatomaceous earth, biocomposites, active containers

## Abstract

Maleinized linseed oil (MLO) has been successfully used as biobased compatibilizer in polyester blends. Its efficiency as compatibilizer in polymer composites with organic and inorganic fillers, compared to other traditional fillers, has also been proved. The goal of this work is to optimize the amount of MLO on poly(lactic acid)/diatomaceous earth (PLA/DE) composites to open new potential to these materials in the active packaging industry without compromising the environmental efficiency of these composites. The amount of DE remains constant at 10 wt% and MLO varies from 1 to 15 phr (weight parts of MLO per 100 g of PLA/DE composite). The effect of MLO on mechanical, thermal, thermomechanical and morphological properties is described in this work. The obtained results show a clear embrittlement of the uncompatibilized PLA/DE composites, which is progressively reduced by the addition of MLO. MLO shows good miscibility at low concentrations (lower than 5 phr) while above 5 phr, a clear phase separation phenomenon can be detected, with the formation of rounded microvoids and shapes which have a positive effect on impact strength.

## 1. Introduction

Natural oils and, in particular, vegetable oils, are currently being widely investigated as they could be a source of a wide variety of new environmentally friendly materials from renewable resources that could positively contribute to sustainable development. In addition, some of these natural vegetable oils cannot be used in the food industry because of regulation restrictions due to their composition and other components. For this reason, some of these vegetable oils are obtained as by-products from other industries, and this contributes to their high worldwide availability, together with their cost-effective price. Recently, selectively modified vegetable oils have been proposed as interesting materials for compatibilization of polymer blends. Other applications of these modified vegetable oils include partially biobased thermosetting resins as an alternative to petroleum-derived resins such as epoxies, which can also be used as matrices in high environmental efficiency green composites. In addition to this, modified vegetable oils are widely used as secondary plasticizers in poly(vinyl chloride)—PVC—to provide increased thermal stability [1,2,3,4,5,6,7,8]. To tailor the desired functionality of a vegetable oil, different chemical modifications have been proposed, including epoxidation, maleinization, acrylation, and hydroxylation, among others.

**Figure 1:**
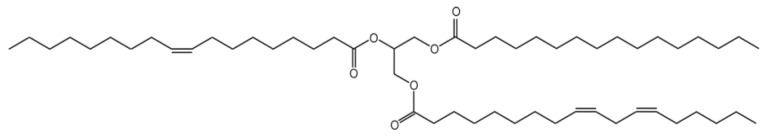
Schematic representation of a triglyceride structure which is the base of vegetable oils, showing different (saturated and unsaturated) fatty acids bonded to a glycerol basic structure through ester bonds.

Vegetable oils are interesting from a chemical point of view because of their triglyceride structure, which consists of a glycerol basic structure which is chemically bonded to different fatty acids through ester bonds. Fatty acids can be saturated as stearic acid (C18:0, which means a chain length of 18 carbon atoms without any unsaturation), palmitic acid (C16:0) or margaric acid (C17:0). These saturated fatty acids are not interesting for chemical modification. Nevertheless, some fatty acids can contain one, two or more unsaturations, thus leading to unsaturated fatty acids such as palmitoleic acid (C16:1), oleic acid (C18:1), linoleic acid (C18:2) or linolenic acid (C18:3), and others. Figure 1 shows a schematic representation of the chemical structure of an unsaturated vegetable oil.

Unsaturations are highly reactive points, such that they represent the base for a chemical modification to provide the desired functionality. Epoxidation is one of the most investigated chemical modification of a vegetable oil. By a simple epoxidation process with peroxoacids derived from, for example, hydrogen peroxide and acetic acid, unsaturations can be converted into oxirane rings [5,9,10,11]. These oxirane rings allow crosslinking in a similar way to a petroleum-derived epoxy resins with different hardener systems [12]. Moreover, oxirane rings increase the polarity of the triglyceride, and this provides good plasticization properties to different polymers. In particular, epoxidized soybean oil (ESBO) and epoxidized linseed oil (ELO) are commercially available as secondary plasticizers for polyvinyl chloride (PVC) but their use has been extended to other polymers such as poly(lactic acid), poly(hydroxybutyrate), and so on [13,14,15,16,17,18]. Other interesting chemical modification of vegetable oils is acrylation, which is carried out on previously epoxidized vegetable oils by reaction with acrylic monomers (acrylic acid, methyl methacrylate, and so on). These acrylic monomers react with the oxirane rings thus leading to increased reactivity to give interesting plasticizer materials or thermosetting resins with a similar behavior to vinyl-ester resins [12,15,19]. On the other hand, vegetable oils can be subjected to a maleinization process, which increases reactivity as well as the above-mentioned methods [20,21,22,23]. This modification is based on the reaction of maleic anhydride with unsaturations, thus leading to anchorage of maleic anhydride in the triglyceride structure [21,24]. Some recent investigations have revealed the interesting plasticization effect of maleinized vegetable oils in some polymers such as poly(lactic acid)—PLA—to increase chain mobility [1,25], but in general, there are few research works on the potential of these biobased materials as additive in plastic formulations. In addition to the plasticization effect of maleinized linseed oil (MLO) on PLA, it has been corroborated the coupling/compatibilizing effect of MLO on PLA composites with diatomaceous earth [26]. For this reason, MLO stands out as an alternative to traditional compatibilizers in the food packaging industry and, in particular, in active packaging, as diatomaceous earth particles are highly porous structures that can be loaded with antioxidants that can be released in a controlled way to increase the shelf life of a product. This approach has given interesting results. For example, Tornuk et al. and Brandelli et al. have reported the use of montmorillonite (MMT) and halloysite nanotubes (HNTs) as carriers for different active principles for active packaging [27,28,29,30,31,32]. Diatomaceous earth (DE) represent an interesting alternative to other clays/nanoclays. From a chemical point of view, DE is composed of amorphous silica, SiO_2_·n H_2_O. From a morphological point of view, it consists hierarchical micro/nanoporous structure. It is this hierarchical porosity that allows the use of DE as carriers for active principles in active packaging. DE is composed of micro-shells of marine unicellular eukaryote organisms in phytoplankton and formed a sediment millions of years ago. Diatom fossilization led to formation of huge diatomaceous earth deposits; therefore, it is an abundant cost-effective product. The main properties of DE have qualities of very low density, porous structure, abrasive, chemical inertness, biocompatible, high absorption capacity, low thermal conductivity, high resistance to acids, and permeability, among others. Currently, diatomaceous earth is widely used as filtration media, for absorption, as a natural insecticide, as functional additives, dental fillings, membranes, and chemical sensors, among other things. When used as natural fillers, DE can provide two different effects: on the one hand, they can provide some reinforcement effect, and on the other hand, they can act as carriers for the controlled release of active principles [33,34,35,36,37]. In a previous work [26], we reported the exceptional performance of maleinized linseed oil as a compatibilizer in PLA/DE composites, compared to other conventional compatibilizers. In this work, the main goal is to optimize the DE/MLO ratio to obtain the best balanced properties on poly(lactic acid)—PLA/diatomaceous earth—DE composites.

## 2. Experimental

### 2.1. Materials

The polymer matrix used in this study was a commercial grade of poly(lactic acid) manufactured and distributed by Nature Works LLC (Minnetonka, MN, USA). This commercial grade was Ingeo Biopolymer 6201D with a melt flow index in the 15–30 g/(10 min) range at 210 °C, which makes it suitable for injection moulding and melt spinning of fibres as well. It is a lightweight material with a typical density of 1.24 g cm^−3^. Regarding diatomaceous earth (DE), this was supplied by ECO-Tierra de diatomeas (Granada, Spain). Table 1 summarizes the composition of this DE.

**Figure 2:**
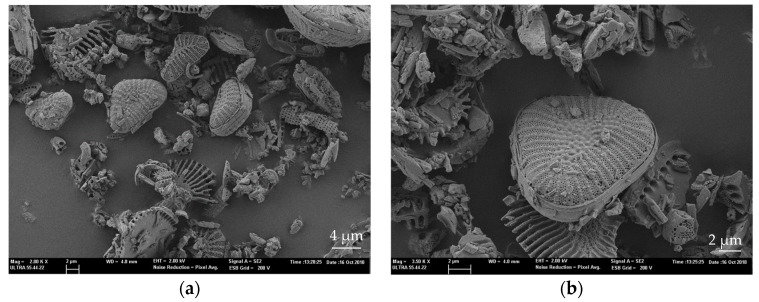
FESEM images of diatomaceous earth at different magnification (**a**) 2000× and (**b**) triangular shape detail at 3500×.

This DE shows different particle sizes and shapes, but triangular shapes with rounded angles are predominant, as can be seen in Figure 2. The average particle size is between 4 and 7 µm. It is worth noting the highly porous structure of these DE particles.

The compatibilizer used in this study was maleinized linseed oil (MLO), which is obtained from the reaction of maleic anhydride (MA) with the unsaturations contained in linseed oil (oleic acid-C18:1, linoleic acid-C18:2 and linolenic-C18:3). This MLO was a commercial-grade VEOMER LIN supplied by Vandeputte (Mouscron, Belgium). Some of its features included a viscosity of 10 dPa s measured at 20 °C and an acid value in the 105–130 mg KOH g^−1^ range.

### 2.2. Manufacturing of PLA/DE Composites Compatibilized with MLO

Table 2 summarizes the compositions and coding of the developed formulations. Prior to any processing, PLA and diatomaceous earth were dried at 60 °C for 18 h to remove the residual moisture.

The above-mentioned compositions were subjected to an initial compounding stage in a co-rotating twin screw extruder from DUPRA S.L. (Alicante, Spain). Different temperatures were selected for the extrusion process by taking into account that the melt peak temperature of PLA is close to 170 °C; therefore, the initial heating stage close to the hopper was set to 165 °C and progressively increased up to 180 °C in the extrusion die. A rotating speed of 20–25 rpm was used. After the compounding stage in a co-rotating twin screw extruder a continuous filament (4 mm diameter) was obtained. This filament was cooled down in air to room temperature (to avoid hydrolysis) and dropped into a shredder manufactured by Mayper (Valencia, Spain) which gave an average pellet size 3 mm in diameter and 2–2.5 mm in height. The pellet of the different composites obtained was then processed by injection moulding process using a Meteor 270/75 from Mateu & Solé (Barcelona, Spain) injection moulding machine. The temperature profile, from the feeding zone to the injection nozzle, was set to: 170–180–190–200 °C. The material was processed with a holding pressure of 75 bar, with an injection time of 8 s in mold and 20 s as cooling time in the open mould.

### 2.3. Characterization and Testing

#### 2.3.1. Thermal and Thermo-Mechanical Characterization

DE can affect thermal behaviour of PLA matrix. For this reason, the effects of DE addition and MLO on thermal transitions of PLA/DE composites were obtained using differential scanning calorimetry (DSC) using a Mettler Toledo 821 calorimeter (Schwerzenbach, Switzerland). A typical procedure is based on the use of a sample weight of about 7–10 mg. Samples were accurately weighed and placed into standard aluminium sealed pans (40 µL). The thermal program was divided into three different stages. The first stage was programmed from 30 °C to 200 °C at a heating rate of 10 °C min^−1^. This stage was applied to remove the previous thermal history which is particularly important in semicrystalline polymers. After this, a cooling stage from 200 °C down to 0 °C at a constant cooling rate of 10 °C min^−1^ was applied. With this stage, samples are subjected to a controlled cooling process which allows further comparisons. Finally, a new heating cycle from 0 °C to 300 °C at a heating rate of 10 °C min^−1^ was applied, and all thermal transitions were obtained in this second heating cycle. To avoid undesired oxidations a nitrogen inert atmosphere (66 mL min^−1^) was used. An important parameter in semicrystalline polymers is the degree of crystallinity (χ_c_) which represents the ratio between the crystalline areas contained in the polymer and the total volume. The degree of crystallinity (χ_c_) was calculated by using the following expression:

(1) $$\chi_{c}=\frac{\Delta H_{m}-\Delta H_{cc}}{\Delta H_{m}^{0}\cdot \left(1-w\right)}\cdot 100\left(\%\right)$$

In this equation, $\Delta H_{m}$ and $\Delta H_{cc}$ (J g^−1^) represent the melt and cold crystallization enthalpies, respectively, while $\Delta H_{m}^{0}$ corresponds to the theoretical melt enthalpy of a fully crystalline PLA, and was taken as 93.0 J g^−1^, as reported in literature [38]. Finally, (1-w) stands for the weight fraction of PLA in the sample without DE or MLO.

Complementary to the characterization of thermal transitions, the thermal stability was evaluated by means of thermogravimetric analysis (TGA) using a TGA/SDTA 851 thermobalance from Mettler Toledo Inc. thermobalance (Schwerzenbach, Switzerland). The selected thermal program was a dynamic heating from 30 °C to 700 °C at a constant heating rate of 20 °C min^−1^ using air atmosphere to simulate more aggressive conditions than using inert atmosphere. The sample weight mass varied in the 8–10 mg range and all the samples had similar dimensions to obtain comparable and reproducible results. Standard alumina crucibles (70 mL) were employed for TGA characterization.

Dynamic mechanical behaviour of PLA/DE composites with different MLO loadings was used to follow the evolution of the storage modulus (*G′*) and the dynamic damping factor (*tan* δ) as a function of increasing temperature. To this end, an AR-G2 oscillatory rheometer from TA Instruments (New Castle, PA, USA), equipped with an environmental test chamber (ETC) and a special clamp device for solid samples, was using in torsion/shear mode. Rectangular samples with dimensions of 40 × 10 mm^2^ and an average thickness of 4 mm were subjected to a temperature sweep from 30 °C up to 140 °C at a heating rate of 2 °C min^−1^. This temperature range was selected because the main thermal transitions of PLA in the solid state, i.e., the glass transition temperature (T_g_) and the cold crystallization occur in this range. Other characteristics of this experiment were defined by a maximum shear deformation (%γ) of 0.1% and a frequency of 1 Hz.

#### 2.3.2. Mechanical Characterization

Mechanical properties of PLA/DE composites with varying MLO loading were obtained from tensile tests following ISO 527-1 in a universal test machine ELIB 30 from S.A.E. Ibertest (Madrid, Spain). The selected conditions for these tests were: load cell of 5 kN, crosshead speed of 10 mm min^−1^. Different tensile properties were obtained and averaged from five different tests for each sample, i.e., tensile strength ($\sigma_{t}$), tensile modulus ($E_{t}$) and elongation at break (%$\varepsilon_{b}$).

Mechanical response of PLA/DE composites in impact conditions were obtained using a Charpy pendulum with a total energy of 1 J from Metrotec S.A. (San Sebastián, Spain) following the guidelines of ISO 179. Five unnotched samples were tested for each formulation, and the impact strength was calculated in kJ m^−2^ by taking into account the cross section of samples.

In addition to the above-mentioned characterization techniques, Shore D hardness was obtained in a durometer 673-D from J. Bot S.A. (Barcelona, Spain), as indicated in ISO 868. In a similar way, hardness was measured in five different samples, and the average values were collected.

#### 2.3.3. Microscopic Characterization

The internal morphology of PLA/DE composites was studied from fractured samples on impact tests. A field emission scanning electron microscope (FESEM) from Oxford Instruments (Abingdon, United Kingdom) working at an acceleration voltage of 2 kV was used. To provide conducting properties to samples and avoid sample charge, all fractured samples were covered with an ultrathin gold-palladium alloy in a Quorum Technologies Ltd. EMITECH model SC7620 sputter coater (East Sussex, UK).

## 3. Results and Discussion

### 3.1. Thermal Properties of PLA/DE Composites with Varying MLO Loading

**Figure 3:**
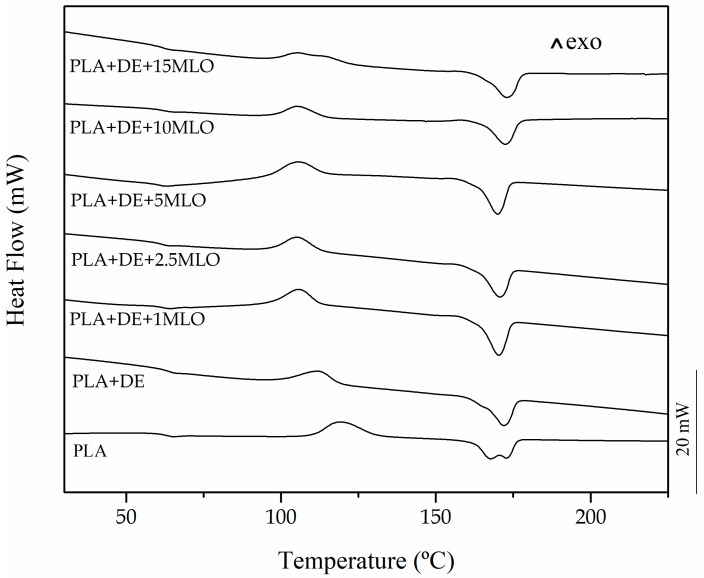
DSC thermograms of neat PLA and PLA+DE composites with different MLO loading (expressed in phr).

A comparative plot of the DSC thermograms of neat PLA and PLA/DE composites with varying MLO content is gathered in Figure 3. A first thermal transition can be seen at around 60 °C that corresponds to the glass transition temperature (T_g_) of PLA. As PLA is highly sensitive to the cooling process, which affects the degree of crystallinity, a cold crystallization peak can be observed with a peak maximum of 119 °C, while this characteristic peak moves down to lower values in PLA/DE composites. The cold crystallization process occurs at lower temperatures with 10 wt% DE. In particular, the peak maximum is displaced to 112 °C. This slight change in the cold crystallization characteristic temperatures is directly related to the fact that DE particles can act as nucleants for crystallization, thus favouring crystallite formation [39,40,41,42]. At higher temperatures, close to 170 °C, an endothermic peak can be observed which is attributed to the melt process of the crystalline fraction in PLA. Table 3 shows the main thermal results obtained from DSC characterization.

Addition of MLO provides a slight decrease in T_g_ of PLA/DE composites. In particular, the maximum decrease is obtained for a MLO loading of 5 phr which gives a T_g_ value of 60.2 °C (3.6 °C lower than PLA/DE composites). This slight decrease in T_g_ is representative for poor plasticization effects, as observed in other polymer systems [1,43,44]. Nevertheless, with regard to the cold crystallization process, a clear decrease in the peak temperature (T_cc_) can be seen from 112 °C (PLA/DE composite) down to values of 105 °C for almost all composites, independently of the MLO loading. MLO favors crystallization due to increased chain mobility. On the other hand, the melt peak temperature of the obtained materials does not change in a remarkable way, with values of about 170 °C, even with increasing MLO content. With regard to normalized enthalpies related to the cold crystallization and melting processes, it is worth noting that they are very useful for making an estimation of the degree of crystallinity (χ) of the PLA/DE composites with increasing MLO content. Neat PLA shows a degree of crystallinity of 9.7%, while the addition of 10 wt% DE leads to increased crystallinity up to values of 15.7% due to the nucleant effect of DE. This is also consistent with the decrease in the cold crystallization peak temperature, as stable crystallites can be obtained at lower temperatures. By adding low MLO loads in the 1–2 phr range, the degree of crystallinity remains almost constant but high MLO loading in the 5–15 phr range, favour the stability of the amorphous PLA domains which is detectable by a decrease in the degree of crystallinity to values of 13%.

**Figure 4:**
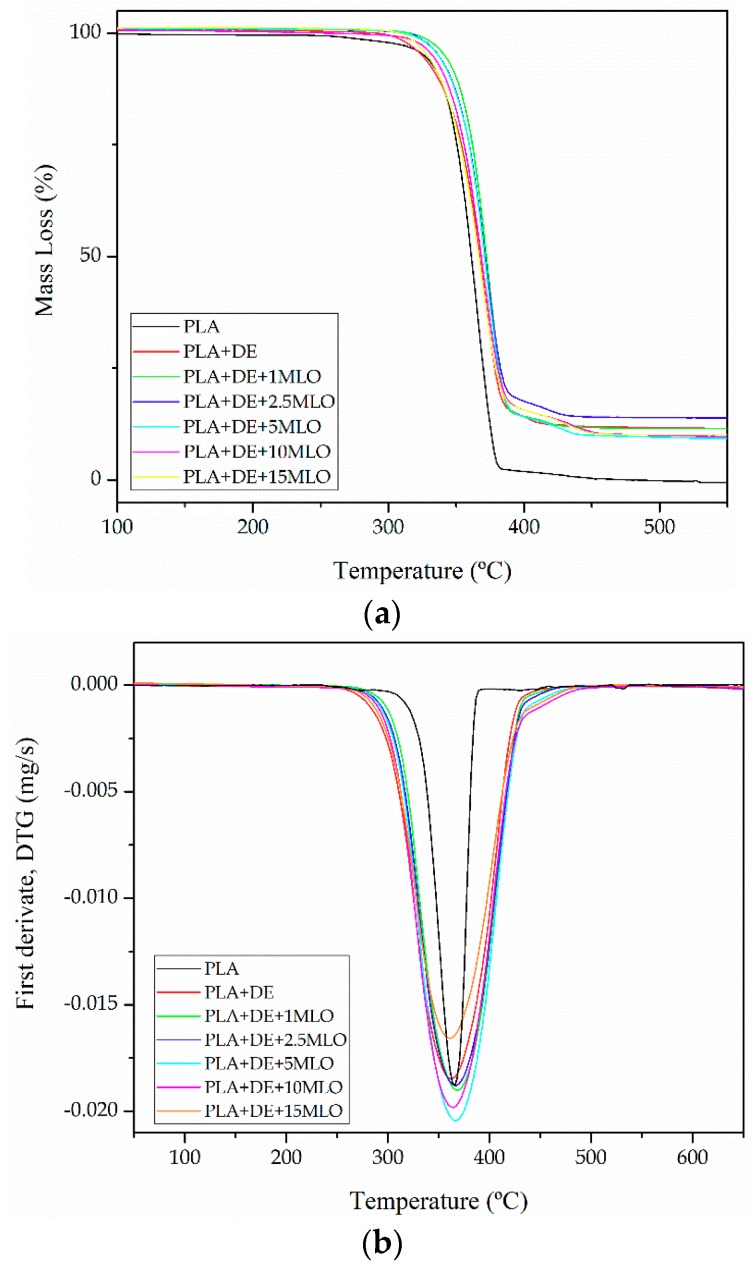
Comparative thermogravimetric (TGA) plots corresponding to PLA/DE composites with varying MLO content: (**a**) TGA thermograms curves and (**b**) first derivative, DTG curves.

With regard to the thermal stability of PLA/DE composites with varying MLO content, Figure 4 shows the thermogravimetric TGA degradation profiles of neat PLA, PLA/DE composite and compatibilized PLA/DE composite with different MLO loadings. As can be observed in Figure 4, all the developed materials in this study show a one-step degradation process. It is important to take into account that the overall thermal stability is related to chemical and physical interactions between the base polymer matrix, PLA, inorganic particles (DE) and MLO [45]. Table 4 shows the main thermal results obtained from TGA analysis.

The simple addition of DE increases the thermal stability of neat PLA. In fact, the onset degradation temperature of PLA (Tonset) changes from 264 °C for neat PLA to 294.3 °C for the PLA/DE composites with 10 wt% DE. These results are in agreement with the work of Carrasco et al., which suggests that addition of small amounts of inorganic materials into a polymer matrix provides increased thermal stability [46]. Moreover, addition of MLO also provides increased thermal stability up to onset degradation temperature values of 315–316 °C for MLO loading in the 1–5 phr range [1]. In fact, the onset degradation temperature for composites with 1–5 phr MLO provides an increase of about 22 °C with regard to the uncompatibilized PLA/DE composite [44]. This phenomenon could be somewhat related to interactions between the PLA chains and the modified vegetable oil [45]. With regard to the residual mass, PLA is almost fully decomposed, while all its composites with DE show residual mass values close to 10% which is in total accordance with the amount of DE filler (10 wt%). DE is composed of inorganic siliceous particles which do not undergo degradation in the temperature range comprised between 30 and 700 °C.

### 3.2. Thermomechanical Properties of PLA/DE Composites with Varying MLO Loading

**Figure 5:**
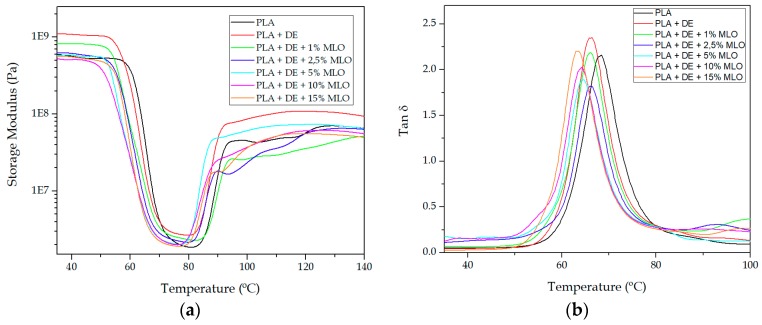
Comparative plot of dynamic mechanical thermal properties of PLA/DE with varying MLO loading as a function of temperature: (**a**) Storage modulus, *G′* and (**b**) dynamic damping factor (*tan* δ).

Figure 5 gathers dynamic mechanical thermal analysis (DMTA) curves corresponding to the evolution of the storage modulus, *G′* (Figure 5a) and dynamic damping factor, *tan* δ (Figure 5b) with temperature. Regarding the storage modulus, below the glass transition temperature (T_g_), all materials show a typical elastic-glassy behaviour with high *G′* values. The uncompatibilized PLA/DE composite shows a *G′* value of 1095 MPa at 40 °C. This *G′* value is remarkably higher than that of the neat PLA (565 MPa at the same temperature). Addition of MLO leads to a decrease in stiffness (lower *G′* values). Thus, the PLA/DE composite with 1 phr MLO shows a *G′* value of 832 MPa at 40 °C. As the MLO content increases, *G′* values show a decreasing tendency as it can be seen in Table 5. This behaviour indicates some plasticization effect of MLO. In fact, some recent research works have suggested that modified triglyceride molecules are placed between polymer chains and, therefore, an increase in chain mobility is achieved, leading to decreased *G′* values. In addition to this internal lubrication effect, MLO molecules increase the free volume this reducing the intermolecular attraction forces between adjacent PLA chains, all this having a positive effect on overall chain mobility [25,47,48]. Above the T_g_, a clear softening occurs, and *G′* values decrease in a remarkable way. As can be seen in Figure 5a, addition of MLO moves the corresponding curves to lower temperatures as MLO enables chain mobility [49,50]. The cold crystallization process can be detected as an increase in *G′* values above the T_g_. As certain temperature is reached, some amorphous areas of PLA tend to re-arrange to form packed structures or crystallites and this increases the density, which is directly related to the stiffness and, consequently, the *G′* is increased [51,52].

Figure 5b shows the damping factor, *tan* δ, for PLA/DE composites with increasing MLO loading. As it can be seen the damping factor is slightly moved towards lower temperatures, compared to neat PLA. Despite it being possible to evaluate the glass transition temperature by using different criteria (*G′*, *G″*, *tan* δ), one of the most widely used is the peak maximum for *tan* δ. By using this criterion, neat PLA shows a T_g_ of 68.1 °C and PLA/DE composite offers a T_g_ of 66.2 °C. As MLO loading increases, a slight decrease in T_g_ can be observed which is in total accordance with previous results obtained by DSC. These T_g_ values are summarized in Table 5.

### 3.3. Mechanical Properties of PLA/DE Composites with Varying MLO Loading

Table 6 summarizes the main mechanical properties of PLA/DE composites with MLO. The only addition of DE (10 wt%) promotes an increase in tensile modulus from 900 MPa (neat PLA) up to 1344 MPa (PLA/DE composite with 10 wt% DE). Nevertheless, both the tensile strength ($\sigma_{t}$) and the elongation at break ($\varepsilon_{b}$) decrease. The tensile strength changes from 65 MPa to 53 MPa with 10 wt% DE and the elongation at break is decreased up to half the value of neat PLA. Dispersion of DE particles leads to a clear embrittlement, as these embedded particles can exert a stress concentration effect, with a subsequent decrease in elongation at break [31].

Addition of MLO on PLA/DE composites provides a decrease in rigidity, as expected. For low MLO loadings in the 1–2 phr range, the tensile modulus is almost constant. Although the average value is slightly lower, if we take into consideration the standard deviation, the change is not significant. Nevertheless, over 5 phr MLO, a clear change in mechanical behaviour can be detected. In particular, the tensile modulus is remarkably decreased. It is worth noting that PLA/DE composites with 15 phr MLO show a tensile modulus of 1075 MPa, which represents a 20% decrease regarding the PLA/DE composite without MLO. The same tendency can be detected for tensile strength. Addition of 5 phr produces a decrease of 10% in tensile strength while above 5 phr, the percentage decrease in tensile strength is comprised between 25–38%. This pronounced change in mechanical resistant properties is inversely related to elongation at break which increases with increasing MLO loading. Uncompatibilized PLA/DE composite is extremely brittle, with an elongation at break of 3.5%. This is slightly increased to values of 5% for low MLO loading but, as expected, above 10 phr MLO, it is possible to obtain a noticeable increase in elongation at break, with values of close to 20% in this range. Some previous works have demonstrated different effects of MLO on PLA and other polyesters. These effects include plasticization, chain extension, branching and, in some cases, some crosslinking [43,47,53]. The plasticization effect has been corroborated by a slight decrease in the glass transition temperature. MLO exerts a lubricant effect on PLA polymer chains and this is responsible for the observed increase in ductility. Changes in stiffness are also related to the ability of the materials to absorb energy. It is important to note that impact strength is related to both ductile and resistant properties. Neat PLA is characterized by an impact strength of 28 kJ m^−2^; due to the stress concentration phenomenon of DE, the PLA/DE composite with 10 wt% DE shows a remarkable decrease in impact strength down to values of 12.4 kJ m^−2^. These results are in total agreement with the dramatic decrease in both tensile strength and elongation at break. As the MLO loading increases, the impact strength improves, and it is worth noting that PLA/DE composites containing 15 phr MLO reach an impact strength of about 22 kJ m^−2^. This is lower than neat PLA, but it is remarkably superior to the uncompatibilized PLA/DE composite. Above 5 phr, MLO can counteract the negative effect of DE on impact strength. Finally, with regard to Shore D hardness, it is worthy to note that although the average values show a slight increase in hardness with DE addition and a decrease with MLO loading, the standard deviation of these values shows that Shore D hardness values remain almost unchanged centred in 80.

### 3.4. Morphological Characterization of PLA/DE Composites with Varying MLO Loading

**Figure 6:**
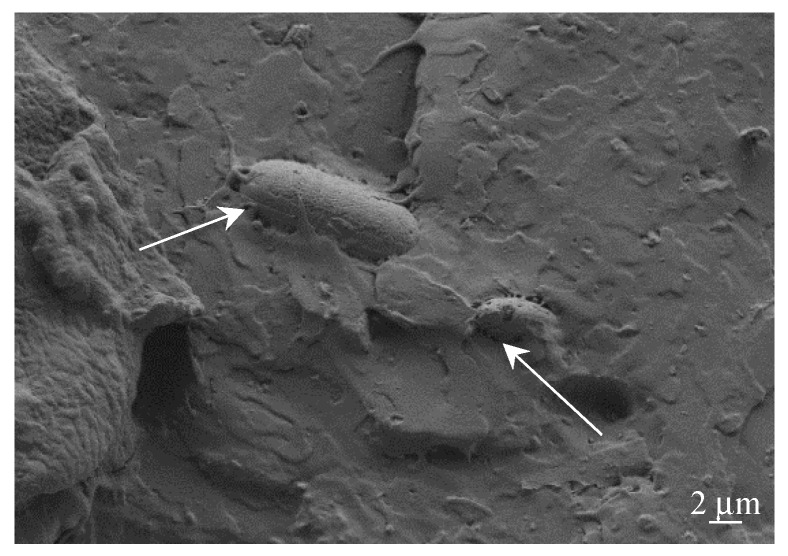
FESEM image (2500×) of the surface of a PLA/DE sample obtained after impact test.

Figure 6 shows a FESEM image corresponding to the fractured surface from the impact test of PLA/DE sample at 2500×. This morphology is characterized by a matrix phase consisting on PLA and a dispersed phase constituted by embedded DE particles. The polymer matrix shows a uniform topography with small steps and, subsequently, low roughness. These are clear evidence of a brittle fracture (or a fracture with very short plastic deformation). In addition, it is possible to identify individual DE particles and the surrounding of these particles show a small gab regarding the polymer matrix. These features are in total agreement with the previous mechanical properties with a decrease in elongation at break and a decrease in tensile strength due to the stress concentration phenomenon provided by uncompatibilized DE particles.

**Figure 7:**
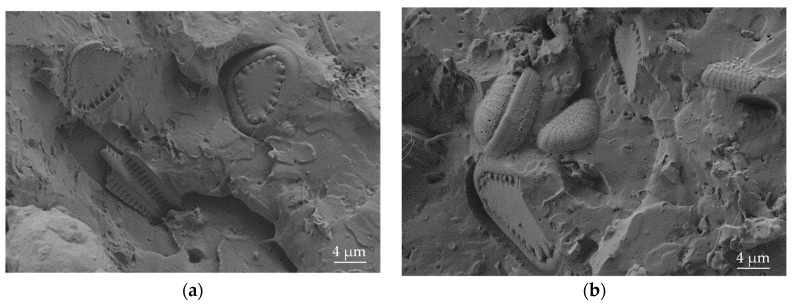

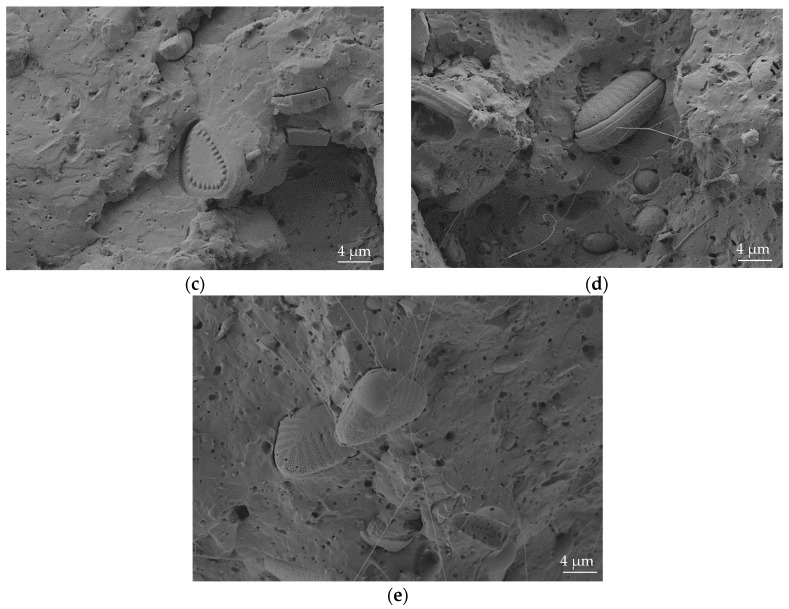
FESEM images (2500×) of the surface of a PLA/DE sample obtained after impact test, with varying MLO loading (in phr): (**a**) 1, (**b**) 2.5, (**c**) 5, (**d**) 10 and (**e**) 15.

Figure 7 shows FESEM images corresponding to PLA/DE composites compatibilized with different MLO loadings. From these images, the PLA matrix and the embedded DE particles (disperse phase) are clearly distinguishable. For low MLO content (1–5 phr), the PLA matrix shows a homogeneous topography, absolutely uniform and smooth. The only difference with the uncompatibilized PLA/DE composite (Figure 6) is that the steps and roughness are rounded and less pronounced, as can be seen in Figure 7a–c. Moreover, it is possible to observe formation of somewhat interphase between PLA and DE particles. In this sense, MLO can act as a compatibilizer of PLA matrix and DE particles due to the tendency of the maleic anhydride group to react with hydroxyl groups in both PLA (terminal groups) and siliceous surface of DE particles. These improvements in the interface phenomena between PLA and DE are responsible for an improvement in ductile properties and, subsequently, on the impact strength, as previously indicated. With respect to PLA/DE composites with high MLO content (10–15 phr), the obtained morphology is somewhat different. The polymeric matrix is less uniform, with a high density of rounded microvoids. In addition, some phase separation can be observed. This indicates that PLA is not completely miscible with MLO. This immiscibility is much more evident with an excess of MLO (see white circles in Figure 7d,e). This restricted miscibility promotes formation of microvoids (see white arrows in Figure 7d,e), as reported in previous works [12,20,25,49,50,54]. In addition to this, it is possible to observe some evidence of plastic deformation (filaments) of the PLA matrix [1]. Therefore, it is possible to conclude that the increase in impact strength is directly related to somewhat interaction between PLA matrix and DE particles.

## 4. Conclusions

This work reports the efficiency of maleinized linseed oil as a biobased compatibilizer in poly(lactic acid)—PLA—composites with diatomaceous earth (DE) at a constant loading of 10 wt%. In particular, the work focuses on the optimization of MLO loading to obtain the most balanced properties on PLA/DE composites. The obtained results show a clear embrittlement of PLA/DE composites without any compatibilizer. The elongation at break is reduced to half the value of neat PLA. On the contrary, the tensile modulus increases as expected. On the other hand, the impact strength of uncompatibilized PLA/DE composites goes down to a value of 12.4 kJ m^−2^, which is remarkably lower than neat PLA (28 kJ m^−2^). Two different levels of effect can be seen depending on the MLO loading. For MLO loadings in the 1–5 phr range, a slight increase in ductile properties can be detected, with a slight decrease in the glass transition temperature (T_g_). Nevertheless, above 5 phr MLO, ductile properties are remarkably improved, and the impact strength increases to values close to 22 kJ m^−2^ which is almost double the value of uncompatibilized PLA/DE composite. The morphology of these composites shows that MLO exerts a compatibilizing effect, bridging the PLA matrix and the DE particles. Therefore, it is possible to conclude that MLO loading in the 10–15 phr range gives optimum and balanced properties for PLA/DE composites without compromising the ecoefficiency of the developed composites.

## Figures and Tables

**Table 1:** Composition of diatomaceous earth used in PLA/DE composites.

Component	Weight Percentage (wt%)
SiO_2_	89.00
Na_2_O + K_2_O	1.88
CaO	6.73
Al_2_O_3_	1.00
Fe_2_O_3_	0.46

**Table 2:** Composition and labelling of PLA/DE composites with different amounts of MLO compatibilizer.

Code	PLA (wt%)	DE (wt%)	MLO (phr) *
PLA	100	0	0
PLA+DE	90	10	0
PLA+DE+1MLO	90	10	1
PLA+DE+2.5MLO	90	10	2.5
PLA+DE+5MLO	90	10	5
PLA+DE+10MLO	90	10	10
PLA+DE+15MLO	90	10	15

* phr represents the weight parts of MLO per 100 weight parts of PLA/DE composites.

**Table 3:** Main thermal parameters of PLA and PLA+DE composites with different MLO loading (expressed in phr) obtained by differential scanning calorimetry (DSC).

Sample	T_g_ (°C)	T_cc_ (°C)	ΔH_cc_ (J g^−1^)	T_m_ (°C)	ΔH_m_ (J g^−1^)	χ_c_ (%)
PLA	63.0	119.5	27.5	169.9	36.5	9.7
PLA-DE	63.8	111.8	19.5	171.3	32.7	15.7
PLA-DE-1MLO	61.8	105.7	21.6	169.7	33.85	14.7
PLA-DE-2.5MLO	61.3	105.2	21.5	170.0	33.6	15.0
PLA-DE-5MLO	60.2	105.5	21.2	169.5	31.2	12.5
PLA-DE-10MLO	62.1	105.5	16.9	172.0	26.9	13.0
PLA-DE-15MLO	61.5	105.6	18.7	172.4	28.1	13.0

**Table 4:** Main thermal parameters of the thermal degradation of PLA/DE composites with varying MLO content obtained by thermogravimetric analysis, TGA.

Sample	T_onset_ (°C)	T_max_ (°C)	Residual Mass (wt. %)
PLA	264.1	366.3	0.16
PLA-DE	294.3	364.3	11.6
PLA-DE-1MLO	316.7	369.6	11.5
PLA-DE-2.5MLO	316.2	367.4	13.8
PLA-DE-5MLO	315.6	367.3	10.0
PLA-DE-10MLO	302.0	364.6	10.3
PLA-DE-15MLO	309.3	361.6	10.2

**Table 5:** Summary of some dynamic-mechanical thermal properties of PLA/DE with varying MLO loading.

Sample	T_g_ (°C) *	Storage Modulus, *G′* (MPa) at 40 °C
PLA	68.1	564.9
PLA-DE	66.2	1095.5
PLA-DE-1MLO	65.9	831.9
PLA-DE-2.5MLO	65.8	624.3
PLA-DE-5MLO	64.5	588.8
PLA-DE-10MLO	64.2	518.4
PLA-DE-15MLO	63.2	545.9

* The T_g_ value has been obtained by using the peak maximum criterium for *tan* δ.

**Table 6:** Summary of mechanical properties of PLA/DE composites with varying MLO loading, obtained from tensile, impact and hardness tests.

Sample	Tensile Modulus, E_t_ (MPa)	Tensile Strength, σ_b_ (MPa)	Elongation at break, ε_b_ (%)	Impact Strength (kJ m^−2^)	Hardness Shore D
PLA	900 ± 75	65 ± 0.5	6.3 ± 0.9	28 ± 3.4	80.5 ± 3.5
PLA-DE	1344 ± 54	53 ± 2.0	3.5 ± 0.3	12.4 ± 2.1	82.3 ± 2.2
PLA-DE-1MLO	1329 ± 70	50 ± 0.5	4.7 ± 0.2	13.4 ± 2.3	82.3 ± 2.1
PLA-DE-2.5MLO	1322 ± 20	46 ± 2.2	4.9 ± 0.3	14.6 ± 2.9	81.9 ± 1.6
PLA-DE-5MLO	1275 ± 48	40 ± 0.6	5.6 ± 0.4	15.6 ± 1.6	82.0 ± 2.2
PLA-DE-10MLO	1161 ± 117	33 ± 0.6	19.5 ± 2	18.4 ± 3.8	81.7 ± 1.6
PLA-DE-15MLO	1075 ± 166	33 ± 0.9	22.8 ± 1.9	21.7 ± 3.6	79.8 ± 2.9
